# Burnout among workers in emergency Departments in Palestinian hospitals: prevalence and associated factors

**DOI:** 10.1186/s12913-017-2356-3

**Published:** 2017-06-15

**Authors:** Motasem Hamdan, Asma’a Abu Hamra

**Affiliations:** Faculty of Public Health, Al-Quds University, East Jerusalem, Palestine

**Keywords:** Burnout, Mbi-Hss, Emergency department, Turnover, Workplace violence

## Abstract

**Background:**

Working in Emergency Departments (EDs) entails high work pressure and stress due to witnessing human suffering and the unpredictable nature of the work. This environment puts personnel at risk of burnout. This analysis aims to assess burnout levels and associated risk factors among health workers in EDs in Palestinian hospitals. Also, it examines the association between burnout and workplace violence, as well as with job turnover.

**Methods:**

Cross-sectional design utilising a self-administered questionnaire was used to collect data from all workers at 14 EDs; 8 from the West Bank and 6 from the Gaza Strip. Burnout was measured using Maslach Burnout Inventory-Human Services Survey.

**Results:**

A total of 444 workers (response rate 74.5%) participated: 161(36.3%) nurses, 142(32.0%) physicians and 141(31.7%) administrative personnel. Results showed high levels of burnout among EDs workers; 64.0% suffered from high emotional exhaustion, 38.1% from high depersonalization and 34.6% from low personal accomplishment. In addition, high levels of emotional exhaustion (72.3%) was significantly prevalent among physicians compared to nurses (69.8%) and administrative workers (51.4%) (*p* < 0.05). In comparison, high levels of depersonalization was significantly prevalent among nurses (48.8%) compared to physicians (32.1%) and administrative workers (31.9%) (*p* < 0.05). However, there were no significant differences in the levels of personal accomplishment burnout among the three groups (*p* > 0.05). Moreover, high degree of burnout was more prevalent among EDs workers in the West Bank than among those working in the Gaza Strip (OR 2.02, 95% CI = 1.11–3.69, *p* = 0.019), and higher among younger workers (aged ≤30 years old) than their older counterparts (OR 2.4, 95% CI = 1.302–4.458, *p* = 0.005). Exposure to physical violence was significantly associated with having a high degree of burnout (OR 2.017 95% CI = 1.121–3.631, *p* = 0.019), but no association was observed with regards to exposure to verbal violence (*p* > 0.05). Finally, burnout was significantly associated with workers’ intention to leave work at EDs (*p* < 0.05).

**Conclusions:**

Burnout is considerably prevalent among EDs’ workers, especially nurses and physicians. Burnout is positively associated with job turnover intention and also with exposure to workplace violence. Therefore, there is a need for prevention and management strategies to address occupational burnout and reduce negative consequences on workers, patients and organisations.

**Electronic supplementary material:**

The online version of this article (doi:10.1186/s12913-017-2356-3) contains supplementary material, which is available to authorized users.

## Background

Occupational burnout is a psychological disorder that is usually prevalent among employees who work in stressful environments [1]. It has been defined by Maslach & Jackson [2] as, "a syndrome of emotional exhaustion, depersonalization and reduced personal accomplishment that can occur among individuals who do ‘people work’ of some kind.” Burnout is common among emergency department (ED) staff [3]. The prevalence of high levels of burnout among ED workers has been attributed to high work pressure, shortages of resources and the nature of care along with witnessing human suffering [4, 5]. Moreover, ED workers are exposed to high risk of workplace violence from patients and visitors [6, 7], such that burnout has been associated with exposure to violence [8, 9]. Evidence shows that ED workers who have been exposed to workplace aggression have a significantly higher percentage of emotional exhaustion and depersonalization [10]. Thus, burnout has potential negative consequences for the healthcare provider (physical and mental), the patient (e.g. quality of care, patient satisfaction) and the health care organisations (e.g. higher absenteeism, turnover, job dissatisfaction, higher costs and financial losses) [1, 11–13]. In specific, turnover intention is more prevalent among employees with high-degree burnout in health care institutions [14–16].

Palestinians in the West Bank (WB) and the Gaza Strip (GS) have been living under prolonged military occupation. This conflict has affected all aspects of people’s lives and still constitutes a real challenge for Palestinians health and the functioning of the health care system. Under circumstances of ongoing conflict, the Palestinian emergency services are carried out under a huge pressure, with insufficient human resources, lack of medication and life-saving equipment, and sometimes, as in the case of Gaza, with frequent electricity cuts. The demand on medical services frequently increases with the flare of Israeli violence against Palestinians, where EDs become overwhelmed with the influx of injuries and traumas. These conditions are probably emotionally and physically challenging for the workers of these departments.

Despite the importance of this issue, evidence about burnout among workers in emergency care services in conflict settings is very limited. The current study complements earlier analysis of workplace violence in Palestinian EDs [7] and aims to assess burnout levels and associated factors among workers in EDs. Moreover, the study aims to examine the association between burnout and workplace violence, as well as with job turnover in EDs.

## Methods

### Design and setting

A cross-sectional design was used to assess the level of burnout among physicians, nurses and admission/registration personnel working in 14 EDs (out of a total of 39 departments) in public and non-governmental hospitals, of which 8 EDs were from the WB and 6 from the GS. These hospitals are the main providers of emergency medical services. At least two hospitals were selected from each of the north, central and south geographic areas of the WB and GS, taking into consideration the representation of the public and non-governmental sectors. It is important to indicate that public hospitals are the main providers of emergency care in Palestine. In addition, Gaza’s hospitals have a larger capacity and number of staff in EDs due to the considerable need for emergency and trauma services in relation to the on-going wars on the GS.

### Population and sample

All employees of the selected hospitals were targeted due to the small number of EDs workers. At the time of study, their number was estimated to be 596 workers, of which 216 were nurses, 201 physicians and 179 administrative/support staff (e.g. registration and reception workers). Those who had an experience of less than one year in the ED and trainees were excluded from the study. Including participants with at least one year experience in EDs originally [7] was intended to calculate the prevalence of workplace violence in the past year.

### Data collection instrument

Data were collected using a self-administered questionnaire (Additional file 1). Burnout was measured through the widely used Maslach Burnout Inventory-Human Services Survey (MBI-HSS) [17]. The Arabic version of MBI-HSS is a validated instrument for measuring burnout among health care workers [18, 19]. The MBI-HSS measures burnout using 22 items grouped in three scales: emotional exhaustion (9 items), depersonalization (5 items) and (reduced) personal accomplishment (8 items). *Emotional exhaustion* measures feelings of being emotionally overextended and exhausted by one’s work; *depersonalization measures* the development of negative, cynical attitudes toward the recipients of one’s services; and *personal accomplishment* measures feelings of competence and successful achievement in one’s work [2, 17]. Participants were asked to answer the MBI items based on how often they experience these feelings on a 7-point Likert scale ranging from 0 (never) to daily (6). The data collection instrument also included sections on the characteristics of the participants and on exposure to workplace violence (physical and non-physical) during the last 12 months. In addition, participants were asked to indicate their intention to quit work at EDs using a five points Likert scale (Very Likely - Likely - Not decided- Less likely - Not at all). The internal consistency of the survey was tested using Cronbach’s alpha coefficient (α = 0.901).

### Data collection

Ethical approval to conduct the study was issued by the institutional review board at Al-Quds University and permissions were obtained from the hospital administrations. Anonymous surveys were distributed with a cover letter that included the aim of the study, survey instructions, as well as an informed consent. Data collection took place in the period between July to September of 2013. Data was collected by trained researchers. Surveys were administered to the participants and collected back in sealed envelopes.

### Statistical analysis

Data entry and statistical analyses were performed with the Statistical Package for Social Sciences (SPSS) software version 19.0. Descriptive statistics were performed for all the study variables. Bivariate analysis using chi-square tests (χ^2^) was used to test for association between MBI subscales and job categories. In addition, prevalence of high degree of burnout was calculated. Whereas high degree of burnout corresponds to having high score on emotional exhaustion, high score on depersonalisation, and low score on personal accomplishment subscales. Odds ratios and 95% CI were used to assess associations between experiencing high degree of burnout and participant characteristics as well as exposure to workplace violence (physical and non-physical). Adjusted odds ratios and 95% CI were used to assess the associations between MBI subscales and intention to quit work in EDs. *P*-value <0.05 was considered as statistical significance in all analyses.

## Results

### Participant characteristics

Table 1 summarizes the characteristics of the participants. The total number of participants was 444 workers, with a response rate of 74.5% from the target population (nurses 80.1%, physicians 65.8%; and administrative personnel 78.8%). Respondents were mostly from the GS (70.5%) and were workers in governmental hospitals (72.7%). Moreover, 68.3% of participants were clinicians (nurses 36.3% and physicians 32.0%), 76.8% males, 52.5% above 30 years of age, 56.3% had experience of less than 5 years in EDs and 67.6% had a bachelor’s degree or higher.

**Table 1:** Demographic and professional characteristics of participants (*n* = 444)

Variables	Number	Percent
Region		
West Bank	131	29.5
Gaza Strip	313	70.5
Sector		
Governmental	323	72.7
Non-governmental	121	27.3
Gender		
Male	341	76.8
Female	103	23.2
Age		
≤ 30 years	211	47.5
> 30 years	233	52.5
Job category		
Physician	142	32.0
Nurse	161	36.3
Administrative/support	141	31.7
Experience in EDs		
< 5 years	250	56.3
5–9 years	111	25.0
≥ 10 years	83	18.7
Level of education		
< Bachelor’s	144	32.4
≥ Bachelor’s	300	67.6

### Prevalence of burnout

Table 2 shows the prevalence of burnout among workers of EDs in Palestinian hospitals. Among them, 64% reported high levels of burnout on emotional exhaustion, 38.1% on depersonalization and 34.6% on the reduced personal accomplishment subscales. Moreover, high levels of emotional exhaustion (72.3%) was significantly prevalent among physicians in comparison to nurses (69.8%) and administrative workers (51.4%) (*p* < 0.05). In comparison, high levels of depersonalization was significantly prevalent among nurses (48.8%) compared to physicians (32.1%) and administrative staff (31.9%) (*p* < 0.05). However, there were no significant differences in the levels of personal accomplishment burnout among the three groups (*p* > 0.05).

**Table 2:** Levels of (MBI) burnout among emergency department by job category

MBI Subscales	Level^a^	Job category				*Χ* ^*2*^ (*p*-value)
		Physician	Nurse	Admin/support	Overall	
		*N*(%)	N(%)	*N*(%)	*N*(%)	
Emotional Exhaustion	Low	10(7.1)	19(11.9)	35(25.4)	64 (14.6)	23.67(*p* < 0.001)
	Moderate	29(20.6)	29(18.2)	32(23.2)	90 (20.5)	
	High	102(72.3)	111(69.8)	71(51.4)	284 (64.8)	
Depersonalization	Low	53(37.9)	50(31.2)	55(39.9)	158 (36.1)	12.53(0.014)
	Moderate	42(30.0)	32(20.0)	39(28.3)	113 (25.8)	
	High	45(32.1)	78(48.8)	44(31.9)	167 (38.1)	
Personal Accomplishment	Low	45(32.1)	51(32.1)	55(39.9)	151 (34.6)	5.39(0.249)
	Moderate	31(22.1)	29(18.2)	32(23.2)	92 (21.1)	
	High	64(45.7)	79(49.7)	51(37.0)	194 (44.4)	

^a^The level of burnout classification is done based on MBI-HSS [12]

χ^2^: Pearson Chi-Square; *p: P*-value

### Factors associated with prevalence of high degree of burnout

Table 3 shows the association between the prevalence of high degree of burnout and participants’ characteristics as well as exposure to workplace violence in the last year. Odds ratios (Table 3) revealed that a high degree of burnout was significantly higher among workers in the WB hospitals than among those who work in the GS (OR 2.02, 95% CI = 1.11–3.69, *p* = 0.019), and higher among younger workers (aged ≤ 30 years old) compared to their older counterparts (OR 2.4, 95% CI = 1.302–4.458, *p* = 0.005).

**Table 3:** Prevalence of high degree of burnout and odds ratios associated with the characteristics of workers and exposure to workplace violence

Variables	High burnout^a^ *N*(%)	OR	95% CI	*p*-value
Region				
West Bank	22(17.2)	2.02	1.11–3.69	0.019
Gaza Strip	29(9.3)	Ref		
Sector				
Governmental	36(11.2)	Ref		
Non-governmental	15(12.7)	1.15	0.6–2.19	0.665
Gender				
Male	43(12.7)	1.69	0.769–3.733	0.168
Female	8(7.9)	Ref		
Age				
≤ 30 years	34(16.2)	2.4	1.302–4.458	0.005
> 30 years	17(7.4)	Ref		
Job category				
Physician	14(9.9)	3.36	0.741–15.261	0.065
Nurse	24(15.0)	5.382	1.233–23.498	
Administrative/support	11(14.7)	Ref		
Experience in EDs				
< 5 years	32(13.0)	1.57	0.666–3.716	0.301
5–9 years	12(10.8)	1.28	0.481–3.413	0.620
≥ 10 years	7(8.6)	Ref		
Level of education				
< Bachelor’s	17(11.8)	1.03	0.553–1.909	0.931
≥ Bachelor’s	34(11.5)	Ref		
Physical violence				
No	25(49.0)	Ref		
Yes	26(51.0)	2.017	1.121–3.631	0.019
Non-physical violence				
No	10(19.6)	Ref		
Yes	41(80.4)	1.792	0.868–3.697	0.115

^a^ High degree of burnout is defined as having high score on emotional exhaustion, high score on depersonalisation, and low score on personal accomplishment

*OR* Odds ratio, *CI* Confidence interval

In addition, burnout was significantly associated with exposure to workplace violence (Table 3). Workers who had been exposed to physical violence in the last year were 2 times more likely to experience a high degree of burnout (OR 2.017 95%CI 1.121–3.631, *p* = 0.019), but no association was observed with regards to exposure to verbal violence (*p* > 0.05).

### Effect of burnout on turnover intention

Table 4 shows the significant association between burnout and workers’ intention to leave work at EDs. EDs workers who had a high level of emotional exhaustion were about 5 times more likely to quit their job than those with a low level of emotional exhaustion (95% CI 2.163–12.194, *p* = 0.001). Also, those who had a high level of depersonalization were about 3 times more likely to quit work in EDs (95% CI = 1.424–5.288, *p* = 0.003) compared to those with a low level. However, there was no significant association between personal accomplishment level and turnover intention (*p* > 0.05).

**Table 4:** Adjusted odds ratios and 95% CI for the effect of burnout syndrome on turnover intention

Burnout subscales	OR^a^	95% CI	*p*-value
Emotional exhaustion			
Low	Ref		
Moderate	2.7	1.009–7.243	0.048
High	5.13	2.163–12.194	0.001
Depersonalization			
Low	Ref		
Moderate	1.77	0.903–3.459	0.096
High	2.74	1.424–5.288	0.003
Personal accomplishment			
Low	Ref		
Moderate	1.04	0.554–1.939	0.910
High	0.994	0.485–2.038	0.987

^a^Adjusted for age, gender, region (West Bank/Gaza Strip)

## Discussion

In the present study, the findings showed that burnout is very high among workers in the EDs of Palestinian hospitals in comparison with evidence from the region [8, 9, 18, 19]. In particular, emotional exhaustion was highly prevalent. Maslach and colleagues (2001) [1] indicated that emotional exhaustion is associated with health problems and can negatively influence the metal health and well-being of personnel, leading to feelings of anxiety, depression and loss of self-esteem. Emotional exhaustion has been attributed to high workload, job stress and lack of control over the work environment [1]. In Palestine, the high levels of emotional exhaustion among ED workers can be explained by the excessive work pressure, which is due to the ongoing conflict in the occupied territories and the consequent large number of injuries and traumas, which require emergency care, as well as the ordinal patients seeking care in these departments. Consequently, workers experience considerable job stressors while dealing with life threatening situations and treating severe war injuries and time pressure. Adding to that, Palestinian hospitals work under difficult conditions and suffer from shortages of human resources, medicines and equipment.

An interesting finding was that despite the extremely difficult work conditions of Gaza hospitals, workers in the WB had higher levels of burnout compared to their colleagues in Gaza. This shows that GS workers are more resilient to work pressure and have stronger capacities to cope with the stressful environment of emergency services.

Consistent with earlier results [5, 9], administrative workers had lower burnout levels than physicians and nurses. Physicians had the highest levels of emotional burnout. This has been attributed to the emotional stress and to the responsibility and accountability of physicians to their patients [20]. In comparison, nurses had a higher depersonalization burnout than physicians and administrative/support workers. This might be due to the fact that nurses have more contact with patients and families compared to other health personnel, and therefore, they are the most to experience depersonalisation. No significant differences were observed between job categories in the personal accomplishment domain and our results were very similar to the findings of Alameddine and colleagues (2011) [9]. Furthermore, our findings showed that high levels of burnout were significantly reported by younger personnel. With time, older workers learn how to manage occupational stressors successfully and become more resilient and adaptive to burnout [21].

Workplace aggression in EDs negatively affects the mental health and well-being of the workers. The psychological and emotional consequences of exposure to workplace violence, such as burnout, anxiety and depression are considerable [10, 22]. In line with earlier studies, our results showed that exposure to workplace violence was significantly associated with increased levels of burnout in the area of emotional exhaustion and depersonalisation [8]. As reported by an earlier study on workplace violence in Palestinian EDs [7], exposure to violence and aggression towards nurses and physicians tends to contribute to the development of a negative and detached response towards the job, such as decreasing contact and time spent with patients and their families. Moreover, the study showed that ED workers significantly expressed feelings of hopelessness, disappointment, fear and anxiety following an exposure to violent incidents.

Job turnover of physicians and nurses is a growing challenge to the functioning of health care systems in many countries. Burnout has an impact on job dissatisfaction and consequently on job turnover [1]. Our findings, in agreement with the results of other studies [9, 11, 15, 23] showed that turnover intention was positively associated with burnout syndrome. ED workers who have suffered from serious burnout on the dimensions of emotional exhaustion and depersonalization were inclined to report higher degrees of turnover intention, but not necessarily on the reduced personal accomplishment dimension.

The current study had many points of strength in that it studied all types of workers in the EDs; included all providers of care in both regions of the country; and had a relatively large sample with a high response rate (74.5%). As for the limitations, this study adopted a cross-sectional design, which makes it difficult to draw proper conclusions on causal effect relationships. Also, the study relied on self-reporting and that might have produced a social desirability bias in the responses of participants.

## Conclusions

Burnout is clearly prevalent among Palestinian EDs workers, especially among nurses and physicians. In particular, emotional exhaustion is of major concern for the mental health and well being of workers. Corroborating earlier studies, high burnout levels have been found to be positively associated with job turnover intention. Therefore, there is need for prevention and management strategies to address occupational burnout. Interventions should include implementing professional education for emergency workers to raise their awareness and to acquire skills to deal with burnout and reduce destructive consequences of burnout for themselves and for their patients. Also, hospitals should ensure social support to workers and enhance the ED resources in order to avoid workload stress. Finally, there is a need for interventions to manage violence and aggression against health care providers in EDs in order to reduce the consequent burnout and job turnover.

## Additional file

Additional file 1: English Translation of the Study Survey. (PDF 600 kb)

## Abbreviations

CI: Confidence interval
ED: Emergency department
GS: Gaza Strip
MBI-HSS: Maslach Burnout Inventory-Human Services Survey
OR: Odds ratio
P: *P*-value
WB: West Bank
χ^2^: Pearson Chi-Square
