# Supplementary material for: Burnout among workers in emergency Departments in Palestinian hospitals: prevalence and associated factors
Source: BMC Health Serv Res. 2017 Jun 15;17:407. doi: 10.1186/s12913-017-2356-3 (PMC5472878; doi:10.1186/s12913-017-2356-3)
Supplement: Additional file 1: — English Translation of the Study Survey. (PDF 600 kb) [file 12913_2017_2356_MOESM1_ESM.pdf]

English Translation of the Study Survey

Assessing Causes and Consequences of Workplace Violence against workers in Palestinian Hospitals Emergency Departments

Dear participant, please read these instructions before you answer the survey

This study is intended to study the prevalence of workplace violence in emergency departments (EDs) of Palestinian hospitals and consequences on workers.

This questionnaire consists of five parts. The first part includes demographic and professional characteristics. The second part deals with workplace physical violence that might have experienced in the emergency department in the past 12 months, while the third part deals with the non-physical violence such as threats, verbal assault, and sexual harassment. Fourth part includes availability of prevention procedures, regulations and policies for violence against workers in the hospital. The last part includes paragraphs on the mental state and the impact of violence on workers in the EDs.

Researchers

Prof. Motasem Hamdan, School of Public Health, Al-Quds University, Jerusalem
Asmaa Abu-Hamra, School of Public Health, Al-Quds University, Gaza Branch

Part one: Demographic and professional characteristics of the respondents:

Please answer the questions by putting (√) in the right place

1.	Hospital name……………………………….					
2.	Sex	□ Male		□Female		
3.	Age	□ Less than 25 years	□ 25-30 years		□ 31-40 years	□ 41 years or older
4.	Job	□Physician	□ Nurse	□Reception/ registration	□Administrative	□Other, Plz. indicate ....…….
5.	Education level	□ Diploma (2 years) or less			□ Bachelors	□ Graduate studies
6.	Experience years in profession……………………					
7.	Experience years in emergency rooms………………………….					

Part two: Physical violence:

Physical assault: Physical assault occurs when a patient, their friend/s, family member/s, other professional/s or work colleague/s is hit, slapped, kicked, pushed, choked, grabbed or otherwise subjected to physical contact intended to injure or harm.

Please answer the following questions by putting (√) for the best answer and only if you had been exposed to physical violence <u>during the last 12 months.</u> <u>If not exposed to physical violence moved to part three.</u>

8.	**In the last 12 months**, have you been physically attacked at your workplace?	□ Once	□ 2-3 times	□4 times or more		
9.	Types of physical violence you had exposed? **(your answer depends on the last incident you had exposed in the last 12 months)**	□Pushing / pulling	□Kicking/ hitting	□Throwing furniture/ equipment	□Weapon	□ other …..…...

10.	**In the last incident**, who physically assaulted you?	□ patient	□ relatives of patient/client		□ colleague	□ other, …………
11.	At which time did it happen?	□ Morning	□ Afternoon		□Night	□ Not sure
12.	Where did the incident take place?	□Reception/ waiting area	□ Examination/ treatment room		□ Corridor	□ another place
13.	Did you receive treatment after the incident and by whom?	□ No need for treatment	□ Needed but didn't receive			□Treated myself
		□ Physician	□ Nurse	□ Psychiatrist		□ Other, ………
14.	In your opinion, what are the reason/ reasons to be physically attacked? ***(Please tick every relevant box)***	□ Waiting time	□ Unmet expectation of patient/ family			
		□ Mental patient	□ Staff attitude with patient			
		□ Anxiety/ fear/ stress	□ Unavailability of needed service/ medicine			
		□ Influence of sickness/ pain	□ Lack of violence prevention methods			
		□ Influence of alcohol/ drugs	□ Don't know			
		□ Other, please specify:______				

<u>Part three: Non-Physical violence</u>

A <u>threat</u> occurs when a patient, their friend/s, family member/s, other professional/s or work colleague/s uses words, gestures or actions with the intent of intimidating, frightening or harming.

<u>Verbal abuse</u> occurs when patient/client, their friend/s, family member/s, other professional/s or work colleague/s using offensive language, yelling or screaming with the intent of offending or frightening you.

<u>Sexual harassment</u> occurs when unwelcome sexual attention from a patients, their friend/s, family member/s, other professional/s or work colleague/s. It includes behaviors such as humiliating or offensive jokes and remarks with sexual overtones, suggestive looks, inappropriate gifts**.**

Please answer the following questions only if you had been exposed to threat or verbal abuse or sexual harassment <u>during the past 12 months</u>. But if you did not expose to any of these types of violence, please go to part four:

15.	**In the last 12 months**, have you been threaten at your workplace?	□ Not exposed	□ One time	□ 2-3 times	□ 4-5 times	□ 6 and more
16.	**In the last 12 months**, have you been verbally abused in your workplace?	□ Not exposed	□ One time	□ 2-3 times	□ 4-5 times	□ 6 and more
17.	**In the last 12 months**, have you been sexually harassed in your workplace?	□ Not exposed	□ One time	□ 2-3 times	□ 4-5 times	□ 6 and more
18.	Please think of **the last time** you were sexually harassed in your place of work. **Who** sexually harassed you?	□Patient	□ Patient relative/ accompany/ visitor		□ Colleague	□ Other ……….
19.	Did you receive treatment after the incident and by whom?	□ No need for treatment	□ Needed but didn't receive			□ Treated myself
		□ Psychiatrist	□ physician		□ nurse	□ Other …………

<u>Part four: Available prevention measures and reporting systems for workplace violence:</u>

Please answer the following questions which about the systems, means available and procedures for preventing and reporting violence against workers in the hospital:

1	Are there violence deterrents available in the emergency department (security, camera, alarm, communication system)?	□_ Yes	□ No
2	Is there a workplace violence policy and program for your hospital to prevent violence against the employees?	□_ Yes	□ No
3	Have you received training or education program in preventing and dealing with workplace violence?	□_ Yes	□ No
4	Is there a system for reporting violence at your hospital?	□_ Yes	□ No

5	Have you reported an incident of workplace violence to management or to your supervisor?	□Yes	□ No (move to NO. 7)	□ Not applicable (move to NO. **9)**
6	Had an action been taken against the assaulter?	□_ Yes		□ No

7	If you did **not** report about the incident to others, why not? ***(Please tick every relevant box)***	□ it was not important	□ afraid of negative consequences	
		□ Felt ashamed	□ did not know who to report to	
		□ Useless (no action would be taken against the assaulter)	□ Other, please specify:______	
8	What are the impact or negative consequences of exposure to violence on you?	□ Minimize time of patient care	□ Avoid taking decision that might involve medical risks	
		□ Minimize communication/ contact with patients/ families/ accompanies	□ Fear and anxiety	
		□ Feel of guilt	□ Feel to revenge	
		□ Hopelessness/ disappointment	□ No impact on me	
9	Do you think to quit work in emergency department during the next 1-3 years?	□ Very likely	□ likely	□ Very likely
		□ Less likely	□ Not at all	

Part five: Mental state and the impact of work on emergency departments workers:

Please answer the following questions by putting (√) on the best answer that expresses how you feel about working in the emergency department:

Items		Daily	Weekly		Monthly		Sometimes yearly	Never
			Several times	Once	Several times	Once		
**1.**	I feel emotionally drained from my work	□	□	□	□	□	□	□
**2.**	I feel used up at the end of the work day.	□	□	□	□	□	□	□

**3.**	I feel fatigued when I get up in the morning and have to face another day on the job	☐	☐	☐	☐	☐	☐	☐
**4.**	Working with people all day is really a strain for me	☐	☐	☐	☐	☐	☐	☐
**5.**	I feel burned out from my work	☐	☐	☐	☐	☐	☐	☐
**6.**	I feel frustrated by my job	☐	☐	☐	☐	☐	☐	☐
**7.**	I feel I’m working too hard on my job	☐	☐	☐	☐	☐	☐	☐
**8.**	Working directly with people puts too much stress on me	☐	☐	☐	☐	☐	☐	☐
**9.**	I feel like I’m at the end of my rope	☐	☐	☐	☐	☐	☐	☐
**10.**	I feel I treat some recipients as if they were impersonal objects	☐	☐	☐	☐	☐	☐	☐
**11.**	I’ve become more callous toward people since I took this job	☐	☐	☐	☐	☐	☐	☐
**12.**	I worry that this job is hardening me emotionally	☐	☐	☐	☐	☐	☐	☐
**13.**	I don’t really care what happens to some recipients	☐	☐	☐	☐	☐	☐	☐
**14.**	I feel recipients blame me for some of their problems	☐	☐	☐	☐	☐	☐	☐
**15.**	I can easily understand how my recipients feel about things	☐	☐	☐	☐	☐	☐	☐
**16.**	I feel I’m positively influencing other people’s lives through my work	☐	☐	☐	☐	☐	☐	☐

**17.**	I feel very energetic	□	□	□	□	□	□	□
**18.**	I can easily create a relaxed atmosphere with my recipients	□	□	□	□	□	□	□
**19.**	I feel exhilarated after working closely with my patients	□	□	□	□	□	□	□
**20.**	In my work, I deal with emotional problems very calmly	□	□	□	□	□	□	□
**21.**	I have accomplished many worthwhile things in this job	□	□	□	□	□	□	□
**22.**	I deal very effectively with the problems of my recipients	□	□	□	□	□	□	□

Finished
Thank you
